# Air pollution and gestational diabetes mellitus: evidence from cohort studies

**DOI:** 10.1136/bmjdrc-2019-000937

**Published:** 2020-03-18

**Authors:** Xingyao Tang, Jian-Bo Zhou, Fuqiang Luo, Yipeng Han, Yoriko Heianza, Marly Augusto Cardoso, Lu Qi

**Affiliations:** 1 Department of Education, Beijing Tongren Hospital, Beijing, China; 2 Department of Endocrinology, Beijing Tongren Hospital, Beijing, China; 3 Department of Epidemiology, Tulane University School of Public Health and Tropical Medicine, New Orleans, Louisiana, USA; 4 Department of Nutrition, School of Public Health, University of São Paulo, São Paulo, Brazil

**Keywords:** air pollution, GDM, accumulated evidence

## Abstract

Exposure to different air pollutants has been linked to type 2 diabetes mellitus, but the evidence for the association between air pollutants and gestational diabetes mellitus (GDM) has not been systematically evaluated. We systematically retrieved relevant studies from PubMed, Embase, and the Web of Science, and performed stratified analyses and regression analyses. Thirteen studies were analyzed, comprising 1 547 154 individuals from nine retrospective studies, three prospective studies, and one case–control study. Increased exposure to particulate matter ≤2.5 µm in diameter (PM_2.5_) was not associated with the increased risk of GDM (adjusted OR 1.03, 95% CI 0.99 to 1.06). However, subgroup analysis showed positive correlation of PM_2.5_ exposure in the second trimester with an increased risk of GDM (combined OR 1.07, 95% CI 1.00 to 1.13). Among pollutants other than PM_2.5_, significant association between GDM and nitrogen dioxide (NO_2_) (OR 1.05, 95% CI 1.01 to 1.10), nitrogen oxide (NO_x_) (OR 1.03, 95% CI 1.01 to 1.05), and sulfur dioxide (SO_2_) (OR 1.09, 95% CI 1.03 to 1.15) was noted. There was no significant association between exposure to black carbon or ozone or carbon monoxide or particulate matter ≤10 µm in diameter and GDM. Thus, systematic review of existing evidence demonstrated association of exposure to NO_2_, NO_x_, and SO_2_, and the second trimester exposure of PM_2.5_ with the increased risk of GDM. Caution may be exercised while deriving conclusions from existing evidence base because of the limited number and the observational nature of studies.

## Introduction

Diabetes mellitus (DM) is a major cause of concern because of its increasing prevalence that has led to a consequential increase in the microvascular as well as macrovascular complications.^1^ Gestational diabetes mellitus (GDM) is a special type of DM characterized by any degree of glucose intolerance with onset, or first recognition during the pregnancy.^2^ It complicates 2%–6% of pregnancies worldwide, and as many as 10%–20% of high-risk pregnancy (body mass index (BMI) >30 kg/m^2^; previous macrosomic baby weighing ≥4.5 kg; personal history of gestational diabetes; family history of gestational diabetes; family history of diabetes) populations.^3^ GDM increases the affected woman’s risk of pre-eclampsia, asymptomatic bacteriuria, pyelonephritis, and cesarean delivery.^4^ Biological factors, such as older age, obesity, and family history, are known to increase the individual’s risk of GDM. However, the exact role and effects of environmental agents in GDM remain unknown.

Air pollution is one of the environmental health risks for GDM.^5^ Many studies have shown that air pollution exposure is related to impaired glucose homeostasis in susceptible populations.^6–8^ Association between air pollution and risk of type 2 diabetes mellitus has been reviewed.^9^ The underlying mechanisms could include endothelial dysfunction, dysregulation of the visceral adipose tissue through inflammation, hepatic insulin resistance, and alterations in autonomic tone that may increase peripheral insulin resistance.^10^ Type 2 diabetes and GDM share common risk factors, and both are characterized by insulin resistance and impaired insulin secretion.^11^

The relationship between air pollutants and GDM has not been studied systematically though a number of related studies have been published.^12–24^ To the best of our knowledge, thus far, there is no available accumulated evidence on their relationship. We therefore systematically identified, and reviewed the epidemiological evidence on the association between air pollutants and GDM.

## Materials and methods

### Study inclusion

The PubMed and Embase databases and Web of Science were searched for relevant studies published until August 2019. Terms used in the search included ‘air pollution’, ‘air pollutant’, ‘particulate matter’, ‘PM_2.5_’, ‘PM_10_’, ‘nitrogen dioxide’, ‘O_3_’, ‘NO_2_’, ‘NO_x_’, ‘SO_2_’, ‘ozone’, ‘soot’, ‘smog’, ‘gestational diabetes’, ‘gestational diabetes mellitus’, ‘GDM’, ‘pregnancy diabetes mellitus’, ‘pregnancy diabetes’, and ‘pregnancy glucose tolerance’ in combination. The search strategy was further supplemented by inspecting the references of the included articles. Two reviewers (XT and YiH) completed the screening independently, and any discrepancies were resolved by discussion. This report was conducted according to the Meta-analysis Of Observational Studies in Epidemiology^25^ and the Preferred Reporting Items for Systematic Reviews and Meta-Analyses^26^ guidelines. Because of reanalysis of published data, ethical approval was not needed for this study.

### Inclusion and exclusion criteria

Studies were considered for inclusion based on the following criteria: (1) the study was an original article published in English; (2) it defined air pollution and GDM status clearly; (3) it measured the outdoor air pollution (ambient, including traffic related); (4) it used physical diagnosis of GDM, if diabetes is diagnosed in the first trimester or early second trimester with the standard diagnostic criteria of a hemoglobin A1c of 6.5% or higher, a fasting plasma glucose of 126 mg/dL or higher, or a 2-hour glucose of 200 mg/dL or higher on a 75 g oral glucose tolerance test, it was considered gestational diabetes^27^; and (5) it provided quantitative measures of association between air pollutants and GDM, and their 95% CIs. Exclusion criteria were as follows: (1) the publication was a review, case report, animal study or letter to the editor, (2) the articles did not clearly define the clinical outcomes, (3) the authors could not provide valid solicited data, and (4) the studies only examined whether the diabetes status would modify the association between air pollution and health outcomes.

For the meta-analysis, only cohort studies about particulate matter ≤2.5 μm in diameter (PM_2.5_), ozone (O_3_), sulfur dioxide (SO_2_), black carbon (BC), nitrogen dioxide (NO_2_), nitrogen oxide (NO_x_), particulate matter ≤10 μm in diameter (PM_10_), and carbon monoxide (CO) were included. We included all studies that quantified these air pollutants as ‘per … μg/m^3^’ or ‘ppb’ or ‘ppm’.

### Data extraction and quality assessment

Two investigators (XT and YiH) independently extracted data from the enrolled studies, using a standard form that included publication year, country of origin, testing method, number of cases, control type, and cut-off value. Two investigators independently assessed the risk of bias for the enrolled studies (XT and FL) using the Newcastle-Ottawa Quality Assessment Scale (NOS) criteria.^28^ Three factors were considered while scoring the quality of included studies: (1) selection, including representativeness of the exposed cohort, selection of the non-exposed cohort, ascertainment of exposure, and the demonstration that at the initiation of the study the outcome of interest was not present; (2) comparability, assessed on the basis of study design and analysis, and whether any confounding variables were adjusted for; and (3) outcome, based on the follow-up period and adequacy of cohorts, and ascertained by independent blind assessment, record linkage, or self-report. We rated the quality of the studies by awarding stars in each domain following the guidelines of NOS. If there was a disagreement, the investigators discussed the research with the other authors to arrive at a consensus.

### Statistical analysis

Heterogeneity and variance between the enrolled studies was evaluated using I^2^ metric, and Tau^2^ respectively. Random effects models were performed to synthesize the association between different air pollutants and GDM in case of I^2^>50%. Random effects models give more weight to smaller studies and have typically wider CIs because the total effect is the average value of the real effect of each study that focuses on the studies with large samples, and pays attention to all included studies in order to balance the effect of each study. Fixed effects models were chosen in case of I^2^≤50%. ORs as the measure of association were pooled across all studies. If studies reported both unadjusted and covariate-adjusted ORs, we included the latter. When risk ratios and incidence ORs were reported, we directly considered them as ORs. For studies providing different methods of air pollution exposure assessments, we chose the results using spatiotemporal models. We used estimates of association and their SEs reported as ‘per 5 µg/m^3^’ of exposure in PM_2.5_, ‘per 10 µg/m^3^’ of exposure in PM_10_, ‘per 0.5 µg/m^3^’ in BC, ‘per 5 ppb’ in O_3_ and SO_2_, ‘per 10 ppb’ in NO_2_ and NO_x_, and ‘per 0.1 ppm’ in CO. We converted other reported quantities or units where necessary. Potential publication bias was evaluated by Egger’s asymmetry test.^29^ P values were two tailed, and p<0.05 was considered statistically significant. Sensitivity analyses were conducted when including at least five data points. The statistical analyses were performed with STATA V.12.0 (StataCorp, College Station, TX, USA).

## Results

### Study selection and study characteristics

**Figure 1:**
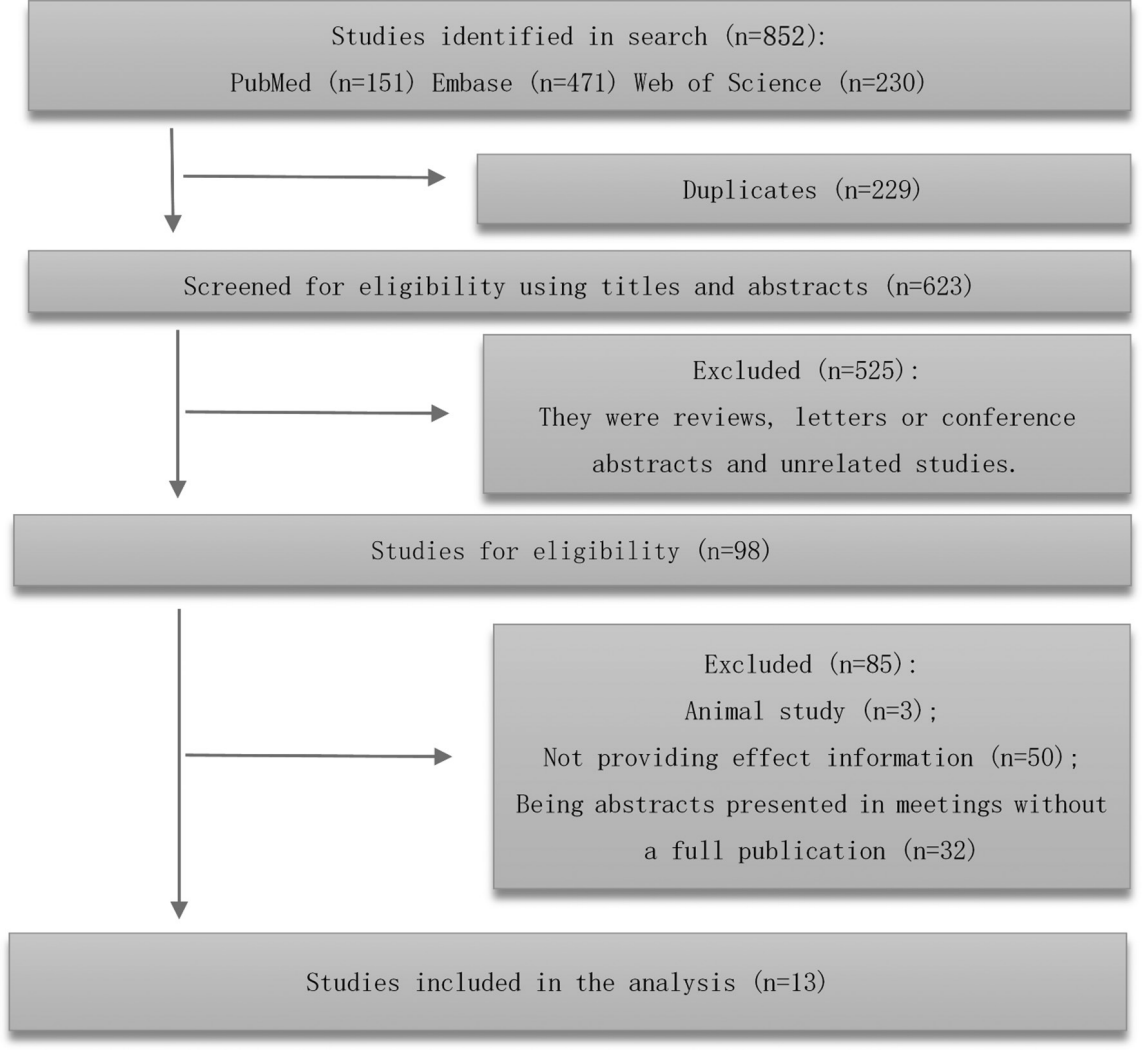
Literature search and selection process.

As per our search strategy, we identified 852 potentially relevant records, of which 229 were duplicate, and thus excluded. The remaining 623 manuscripts were subject to title and abstract screening. Further, 525 publications were removed as they were reviews, letters or conference abstracts or unrelated studies. Therefore, 98 articles were eligible for full-text review and data assessment (figure 1). Finally, 85 articles were excluded for other reasons (animal studies (n=3), unable to extract information (n=50), and lack of full publication (n=32)). The remaining 13 studies were enrolled in the meta-analysis^12–24^ out of which three were prospective cohort studies,^13 19 22^ nine were retrospective cohort studies^12 14–18 20 23 24^ and one was a case–control study.^21^ Seven studies were on PM_2.5_,^12–15 20 23 24^ four studies were on O_3_,^15 18 20 24^ three studies were on PM_10_,^18 20 24^ while two studies on each of the following pollutants were included: SO_2_^18 20^; NO_x_^18 20^; CO; BC^12 13^; and NO_2_.^23 24^Tables 1 and 2 provide an overview of the 13 enrolled studies. Online supplementary table S1 summarizes the data reported in these studies as synthesized in meta-analyses.

10.1136/bmjdrc-2019-000937.supp1 Supplementary data

**Table 1:** Characteristics of the studies on the relationship between air pollutant and gestational diabetes mellitus

Source	Location	Years of study	Study design and duration of follow-up	Population (n) and age (years) of participants	NOS
Choe *et al*^12^	Rhode Island, USA	2002–2012 (excluded July 2004 to December 2005)	Retrospective cohort study	n=61 640 mother–infant pairs, singleton births to mothers aged 18 years or older and residing in Rhode Island during the study period	7
Fleisch *et al*^13^	Boston, Massachusetts, USA	1999–2002	Prospective cohort study	n=2093 second-trimester pregnant women without known diabetes	7
Fleisch *et al*^14^	Boston, Massachusetts, USA	1 January 2003 to 31 December 2008	Retrospective cohort study	n=159 373 primiparous women during the study period without pre-existing diabetes	7
Hu *et al*^15^	Florida, USA	1 January 2004 to 31 December 2005	Retrospective cohort study	n=410 267 women who gave birth in Florida during the study period and without non-singleton deliveries, previous preterm births, or pre-pregnancy diabetes mellitus	8
Lu *et al*^16^	Chiayi City, Taiwan	2006–2014	Retrospective cohort study	n=3589 non-diabetic pregnant women during the study period	7
Malmqvist *et al*^17^	Scania, Sweden	1999–2005	Retrospective cohort study	n=81 110 women who had singleton deliveries during the study period	8
Pan *et al*^18^	Taiwan	2004–2005	Retrospective cohort study	n=19 606 women were included after the exclusion criteria were applied	8
Pedersen *et al*^19^	Danish National Birth Cohort	1997–2002	Prospective cohort study	n=72 745 singleton pregnancies without hypertension, pre-existing chronic hypertension, and diabetes before pregnancy	7
Robledo *et al*^20^	USA	2002–2008	Retrospective cohort study	n=219 952 singleton deliveries to mothers without pregestational diabetes	8
Shen *et al*^21^	Taiwan	2006–2013	Case–control study	n=6717 mothers as the cases of newly diagnosed GDM n=6717 control mothers were selected	8
van den Hooven *et al*^22^	Rotterdam, Netherlands	2002–2006	Prospective cohort study	n=7399 pregnant women who had a delivery date in the study period, 21–38 years	8
Choe *et al*^23^	New York City	2008–2010	Retrospective cohort study	n=256 372 deliveries without non-singleton births, reporting smoking during pregnancy and mothers with pre-existing diabetes	8
Jo *et al*^24^	Kaiser Permanente Southern California (KPSC) hospitals	1 January 1999 to 31 December 2009	Retrospective cohort study	n=239 574 pregnancies without pre-existing diabetes	8

GDM, gestational diabetes mellitus; NOS, Newcastle-Ottawa Quality Assessment Scale criteria.

**Table 2:** Air pollutant exposure and outcome definitions of studies included

Source	Outcome	Definition of outcome	Exposure	Definition of exposure	Exposure estimates
Choe *et al*^12^	GDM	Birth certificate data and ICD-9648.8x were listed, and absent otherwise.	PM_2.5_, black carbon	PM_2.5_ and black carbon from spatiotemporal models.	Mean±SD First trimester PM_2.5_: 9.7±1.9 µg/m^3^; second trimester PM_2.5_: 9.6±1.9 µg/m^3^ Third trimester PM_2.5_: 9.5±2.1 µg/m^3^; first trimester black carbon: 0.5±0.1 µg/m^3^; second trimester black carbon: 0.5±0.1 µg/m^3^; third trimester black carbon: 0.5±0.1 µg/m^3^
Fleisch *et al*^13^	GDM	Failed GCT(1) with ≥2 high values on the OGTT(2).	PM_2.5_, black carbon, traffic exposure	PM_2.5_ and black carbon from central sites within 40 km of residence. PM_2.5_ and black carbon from spatiotemporal models. Neighborhood traffic density [(vehicles/day) × km] within 100 m.	Mean±SD From central sites: PM_2.5_: 10.9±1.4 µg/m^3^; black carbon: 0.9±0.1 µg/m^3^ From spatiotemporal models: PM_2.5_: 11.9±1.4 µg/m^3^; black carbon: 0.7±0.2 µg/m^3^ Traffic density: 1621±2234 (vehicles/day × km)
Fleisch *et al*^14^	GDM	Failed GCT with ≥2 high values on the OGTT.	PM_2.5_, traffic exposure	PM_2.5_ from spatiotemporal models. Neighborhood traffic density [(vehicles/day) × km] within 100 m.	Mean±SD First trimester PM_2.5_: 10.4±1.7 µg/m^3^; second trimester PM_2.5_: 10.4±1.7 µg/m^3^ Traffic density: 1317±2025 (vehicles/day × km)
Hu *et al*^15^	GDM	According to the American Diabetes Association 2003, failed GCT with ≥2 high values on the OGTT.	PM_2.5_, O_3_	Air pollution exposure data were obtained from the US EPA and CDC’s National Environmental Public Health Tracking Network (2003–2005) (US EPA 2014)	Mean±SD Trimester 1 PM_2.5_: 9.73±2.07 µg/m^3^; O_3_: 37.20±6.04 ppb Trimester 2 PM_2.5_: 9.88±2.06 µg/m^3^; O_3_: 37.54±6.10 ppb Full pregnancy PM_2.5_: 9.93±1.67 µg/m^3^; O_3_: 37.40±4.10 ppb
Lu *et al*^16^	GDM	A woman with a positive GCT and two or more abnormal 100 g OGTT values.	PM_2.5_, SO_2_, NO_x_, CO, O_3_	The exposure assessment of this study based on data from a single fixed-site monitoring station (Chiayi station).	Mean±SD 3 months pre-pregnancy PM_2.5_: 44.38±12.09 µg/m^3^ First trimester PM_2.5_: 43.52±12.87 µg/m^3^; second trimester PM_2.5_: 41.20±13.43 µg/m^3^
Malmqvist *et al*^17^	GDM	GDM as defined in the Swedish Medical Birth Registry.	NO_x_, traffic exposure	Monthly and trimester means of NO_x_ assigned by dispersion modeling at a spatial resolution of 500×500 m throughout the pregnancy. Traffic density within a 200 m radius.	Quartiles of NO_x_ exposure (μg/m^3^): Q1: 2.5–8.9; Q2: 9.0–14.1; Q3: 14.2–22.6; Q4: >22.7 Categories of traffic density within 200 m (vehicles/min): 1: no road; 2: ＜2; 3: 2–5; 4: 5–10; 5: ＞10
Pan *et al*^18^	GDM	According to the American Diabetes Association criteria, had two of the abnormal values on the OGTT.	PM_10_, CO, NO_x_, SO_2_, O_3_	Collected from 77 fixed-site air monitoring stations in Taiwan during 2004–2006.	Mean±SD PM_10_ (μg/m^3^): first trimester: 61.4±18.3; second trimester: 61.2±17.2; third trimester: 62.2±19.5 CO (ppm): first trimester: 0.6±0.1; second trimester: 0.6±0.1; third trimester: 0.6±0.2 NO (ppb): first trimester: 6.5±3.3; second trimester: 6.9±3.1; third trimester: 6.9±3.2 NO_2_ (ppb): First trimester: 20.2±5.3; second trimester: 19.8±5.5; third trimester: 19.1±5.9 NO_x_ (ppb): first trimester: 26.6±7.8; second trimester: 26.5±7.8; third trimester: 25.7±8.2 SO_2_ (ppb): first trimester: 4.5±1.8; second trimester: 4.8±1.7; third trimester: 4.9±1.7 O_3_ (ppb): first trimester: 25.8±3.8; second trimester: 25.6±3.2; third trimester: 25.5±3.7
Pedersen *et al*^19^	GDM	Self-reported, physician-diagnosed GDM.	NO_2_, noise from road traffic (Lden) exposure	NO_2_ was using the advanced AirGIS dispersion model. Road traffic noise was using SoundPLAN based on the Nordic prediction method.	First trimester：NO_2_ (μg/m^3^): 11.5 (5.8, 27.4); road traffic noise (dB): 57.5 (49.3, 69.8); railway noise (dB): 51.3 (31.1, 68.6)
Robledo *et al*^20^	GDM	GDM was recorded in the medical record or discharge records (code 648.8) using the International Classification of Diseases, Ninth Revision.	PM_10_, PM_2.5_, SO_2_, O_3_, CO, NO_x_	Using a modified Community Multiscale Air Quality (CMAQ) model version 4.7.1.	IQR Preconception PM_2.5_ (μg/m^3^): 5.54; PM_10_ (μg/m^3^): 6.3; SO_2_ (ppb): 3.30; NO_x_ (ppb): 28.55; O_3_ (ppb): 12.33; CO (ppm): 0.26 First trimester PM_2.5_(μg/m^3^): 5.28; PM_10_ (μg/m^3^): 6.32; SO_2_ (ppb): 3.31; NO_x_ (ppb): 30.21; O_3_ (ppb): 12.36; CO(ppm): 0.26
Shen *et al*^21^	GDM	International Classification of Diseases, Ninth Revision, Clinical Modification (ICD-9-CM) code: 648.0 or 648.8.	PM_10_, PM_2.5_, SO_2_, O_3_, CO, NO_2_	Collected from 76 fixed-site air quality monitoring stations supervised by the Taiwan Environmental Protection Agency during 2005–2013.	
van den Hooven *et al* 22	GDM	GDM diagnosed according to the Dutch midwifery and obstetric guidelines.	Traffic exposure	Distance-weighted traffic density (DWTD) within a 150 m radius around residence (vehicles/24 hours × m); proximity to a major road (>10 000 vehicles/day).	Median (P25–P75) DWTD (vehicles/24 hours × m): 5.5×10^5^ (1.6×10^5^−1.2×10^6^) Proximity to a major road (m): 140 (74–225)
Choe *et al*^23^	GDM	ICD-9-CM code: 648.8.	PM_2.5_, NO_2_	Air pollution samples were collected at 150 monitoring sites in each of the four seasons for one 2-week session and in every 2 weeks at five reference locations to track city-wide temporal variation.	Mean±SD Trimester 1 PM_2.5_: 12.0±2.5 µg/m^3^; NO_2_: 27.9±6.3 ppb Trimester 2 PM_2.5_: 11.9±2.4 µg/m^3^; O_3_: 27.9±6.3 ppb
Jo *et al*^24^	GDM	Based on laboratory values confirming a plasma glucose level of 200 mg/dL or higher on the glucose challenge test or at least two plasma glucose values meeting or exceeding the following values on the 100 or 75 g oral glucose tolerance test.	PM_2.5_, PM_10_, NO_2_, O_3_	Distance-weighted monthly average from four closest monitoring stations within 50 km, except for geocoded locations within 0.25 km of a monitor.	Mean±SD PM_2.5_: 18.2±5.5 µg/m^3^; PM_10_: 38.4±10.9 µg/m^3^; NO_2_: 25.8±8.2 ppb; O_3_: 41.3±7.6 ppb

(1) Glucose change test: serum glucose 1 hour after a non-fasting 50 g oral glucose load. (2) Oral glucose tolerance test: serum glucose 3 hours after a fasting 100 g glucose load.

CDC, Centers for Disease Control and Prevention; CO, carbon monoxide; EPA, Environmental Protection Agency; GCT, glucose change test; GDM, gestational diabetes mellitus; NO, nitric oxide; NO_2_, nitrogen dioxide; NO_x_, nitrogen oxide; O_3_, ozone; OGTT, oral glucose tolerance test; PM_10_, particulate matter ≤10 μm in diameter; PM_2.5_, particulate matter ≤2.5 μm in diameter; SO_2_, sulfur dioxide.

### Quality assessment

Quality assessment using the NOS evaluation tool resulted in high ratings for all the 13 studies (score 7 or 8) (online supplementary table S2).

### Meta-analysis results

There were 13 sets of data on PM_2.5_ (Q=106.07, I^2^=88.7%, p=0.000), 8 sets of data on O_3_ (Q=344.11, I^2^=98.0%, p<0.001), 6 sets of data on PM_10_ (Q=8.91, I^2^=43.9%, p=0.113), 4 sets of data on each of the following: NO_2_ (Q=17.50, I^2^=82.9%, p=0.001), SO_2_ (Q=4.26, I^2^=29.6%, p=0.234), CO (Q=7.08, I^2^=57.7%, p=0.069), NO_x_ (Q=7.12, I^2^=57.9%, p=0.068), and 3 sets of data on BC (Q=0.34, I^2^=0.0%, p=0.562). As per the heterogeneity, the random effects model was selected for analysis of PM_2.5_, O_3_, NO_2_, CO, and NO_x_, while the fixed effects model was chosen for SO_2_, PM_10_, and BC.

**Figure 2:**
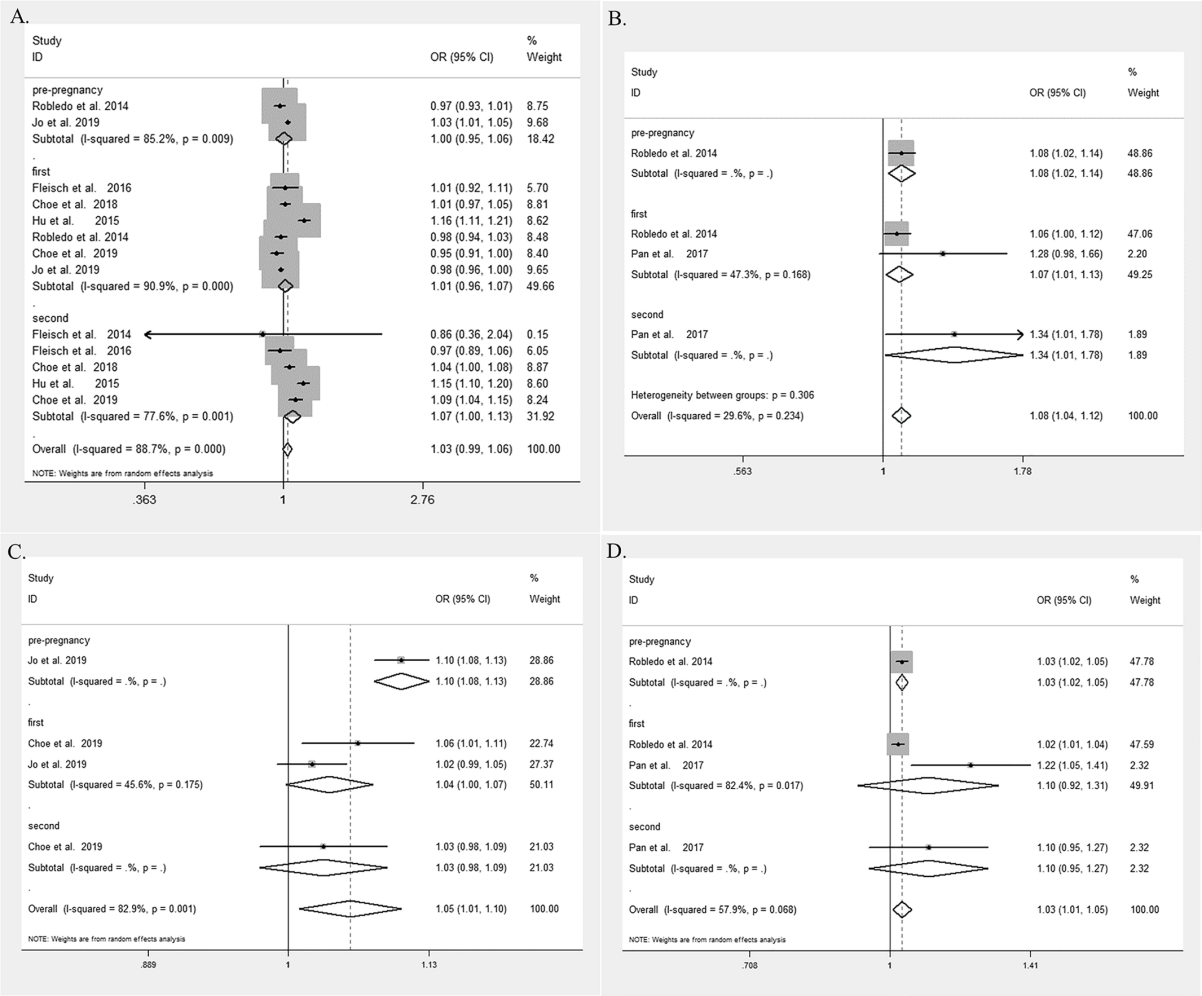
Forest plot and pooled estimates of the association between exposure to A) particulate matter ≤2.5 μm in diameter (PM_2.5_), B) sulfur dioxide (SO_2_), C) nitrogen dioxide (NO_2_) and D) nitrogen oxide (NO_x_) with risk of gestational diabetes mellitus (GDM). Pre-pregnancy, the exposure to PM2.5 was measured before pregnancy; first, the exposure to PM2.5 was measured during the first trimester; second, the exposure to PM2.5 was measured during the second trimester. GDM, gestational diabetes mellitus; NO_2_, nitrogen dioxide; NO_x_, nitrogen oxides; OR, odds ratio; PM2.5, particulate matter ≤ 2.5 μm in diameter; SO2, sulfurdioxide.

The statistically significant pooled effect value was absent in the relationship between PM_2.5_ and GDM (Z test, Z=1.55, p=0.122, the combined OR 1.06, 95% CI 0.99 to 1.03). We further performed the subgroup analysis for PM_2.5_ exposure in the different periods, including the pre-pregnancy, the first trimester and the second trimester. Subgroup analysis revealed that the above non-significant association persisted in both the pre-pregnancy and the first trimester (the overall OR of 1.00 (95% CI 0.95 to 1.06) and 1.01 (95% CI 0.96 to 1.07), respectively). However, in the second trimester, exposure to PM_2.5_ was associated with the increased risk of GDM (Z=2.11, p=0.035, the overall OR=1.07, 95% CI 1.00 to 1.13) (figure 2A).

The significant relationship of exposure to SO_2_ with increased risk of GDM was noted (Z=3.83, p<0.001, the overall OR=1.08, 95% CI 1.04 to 1.12). In the subgroup analysis, the positive association was consistently observed in the pre-pregnancy, the first trimester, and the second trimester (the overall OR of 1.08 (95% CI 1.02 to 1.14), 1.07 (95% CI 1.01 to 1.13), and 1.34 (95% CI 1.01 to 1.78), respectively) (figure 2B).

There was a statistically significant correlation between exposure to NO_2_ and the increased risk of GDM (Z=2.40, p=0.016, the overall OR=1.05, 95% CI 1.01 to 1.10). In the subgroup analysis, the same correlation was persistent in the pre-pregnancy and the first trimester subgroups (pooled OR=1.10 (95% CI 1.08 to 1.13) and 1.04 (95% CI 1.00 to 1.07), respectively) (figure 2C).

Exposure to NO_x_ was also related to an increased risk of GDM (Z=2.62, p=0.009, the overall OR=1.03, 95% CI 1.01 to 1.06). In the pre-pregnancy subgroup, a positive association was noted between the exposure to NO_x_ and GDM (Z=3.96, p=0.000, the overall OR=1.03, 95% CI 1.02 to 1.05). However, in the first trimester, and the second trimester subgroups, the association was missing (first trimester, Z=1.06, p=0.287, the overall OR=1.10, 95% CI 0.92 to 1.31 and second trimester, Z=1.28, p=0.202, the overall OR=1.10, 95% CI 0.95 to 1.27) (figure 2D).

The non-significant relationship between BC and GDM was obtained (Z=1.13, p=0.257, the overall OR=1.02, 95% CI 0.99 to 1.05) (online supplementary figure S1A). Similar results were observed in CO, O_3_, and PM_10_ (Z=0.88, p=0.380, the overall OR=1.01, 95% CI 0.99 to 1.03; Z=0.69, p=0.489, the overall OR=1.01, 95% CI 0.98 to 1.04; Z=0.53, p=0.595, the overall OR=1.00, 95% CI 0.99 to 1.01, respectively) (online supplementary figure S1B,C).

### Sensitivity analysis

Sensitivity analyses of PM_2.5_, PM_10_, and O_3_ were performed through single elimination of studies. The sensitivity analyses between the exposures to PM_2.5_, PM_10_, and O_3_ and the risk of GDM indicated no significant change in results.

### Publication bias

According to the Cochrane Handbook version 5.1.0,^30^ as a rule of thumb, tests for funnel plot asymmetry should be used only when there are not too few research included in the meta-analysis, because when there are fewer studies, the power of the tests is too low to distinguish chance from real asymmetry. Therefore, we restricted this analysis to PM_2.5_, O_3_, and PM_10_, no significant bias exists among the studies by Egger’s test. The funnel figure of these studies showed a symmetrical inverted distribution that was consistent with the results of Egger’s test (online supplementary figure S2).

## Discussion

In this study, we carried out the accumulated evidence to explore the relationship between air pollutants and GDM from observational studies. Results indicated that exposure to PM_2.5_ in the second trimester, and exposures to SO_2_, NO_2_ and NO_x_ were significantly associated with the increased risk of GDM.

In the current analysis, the relationship of PM_2.5_ and risk of GDM was observed only in the second trimester, but not in the pre-pregnancy or the first trimester. This is consistent with the results of a prior study that suggested PM_2.5_ may affect glucose homeostasis only during the second trimester of pregnancy.^23^ Additionally, Fleisch *et al*^13^ found that women with the highest quartile exposure (12.8–15.9 µg/m^3^) to PM_2.5_ during the second trimester had a 2.63 (95% CI 1.15 to 6.01) times higher risk of having impaired glucose tolerance (IGT) than the women who had first quartile exposure. In another study, Fleisch *et al*^14^ noted that women younger than 20 years had 1.36 higher odds of GDM (95% CI 1.08 to 1.70) for each interquartile increment in PM_2.5_ exposure than the older women, at the second trimester. O_3_ was the other air pollutant that showed significant association with GDM in our analysis, consistent with Robledo *et al*,^20^ who found significant associations of GDM with interquartile increment in the preconception (5.37 ppb) and the first trimester (3.31 ppb) periods, with ORs of 1.05 (95% CI 1.01 to 1.09) and 1.04 (95% CI 1.01 to 1.08). A previous study noted increased risks of GDM in relation to nitric oxide exposures,^18^ while our study documented a significant association between NO_2_ and NO_x_ exposure with the risk of GDM.

The possible mechanisms underlying the associations between air pollutants and GDM are still unclear. Several different aspects were raised by many researchers based on their opinions, including inflammation (adipose tissue inflammation,^31^ peripheral inflammation,^32^ systemic inflammation which is indicated by elevated serum C-reactive protein^33^ and cytokines^34^), oxidative damage,^35^ direct endothelial dysfunction,^36^ and dyslipidemia.^37^

PM_2.5_ was considered to initiate toxic effects and stimulate the production of free radicals or reactive oxygen.^38^ Levels of oxidative stress biomarkers, glutathione peroxidase and malonic dialdehyde, for instance, vary after PM_2.5_ exposure.^39 40^ Moreover, PM_2.5_ exposure during pregnancy can downregulate the expression of glucose transporter 2 in pancreatic β-cells and thereby yield glucose intolerance in GDM rats.^41^ Similarly, possible mechanisms linking insulin resistance with exposure to PM_2.5_ have been demonstrated by several human studies and are recognized as one of the important underlying metabolic conditions contributing to the development of GDM.^42^ The observation that O_3_-induced insulin resistance was associated with neuronal activation and sympathetic stimulation has been found by Bass *et al*.^43^ The other opinion shows that O_3_ may damage the β-cells of the pancreas, according to which O_3_ is known to alter T-cell-dependent immune response,^44^ leading to the reduced insulin secretion.^45^ For the SO_2_, studies also showed similar mechanisms, such as inflammation^46^ and dysfunction of pancreatic β-cells.^47^ It has been argued that NO_2_ and NO_x_ can lead similar inflammation responses to those of particulate matter and O_3_.^48^

The strengths of our study included the adjustment for multiple confounders including geographic, sex, BMI, smoking, alcohol consumption, socioeconomic status, and age variables that affected the individual studies, but were reduced by our study design. Further, our meta-analysis is the most recent that comprehensively, critically, and quantitatively assesses the association between air pollutants and gestational diabetes.

Our study had the following limitations. (1) All included studies were observational studies, thus, the causal effect between air pollutants and GDM could not be described. (2) The high heterogeneity identified for some of the pollutants may be due to differences in race, blood glucose measurement, and pollutant concentrations in different regions. (3) This article analyzed respectively the relationship between eight different air pollutants (PM_2.5_, O_3_, SO_2_, NO_2_, NO_x_, CO, PM_10_, and BC) with GDM. Besides these eight kinds of air pollutants, there are also some other pollutants that may influence the risk of GDM.^18^ (4) In our daily life, different kinds of air pollutants are mixed and it is impossible to distinguish them from each other. The influence of the mixed air pollutants could not be analyzed because of the diversity of methods that researchers chose in individual studies. (5) Most studies were performed during the first and second trimesters, however, only few studies were performed before the conception. It was thus difficult to perform analyses during the preconception stage. (6) In addition to concentration of outdoor air pollutants, the distance from the main traffic road and noise, active and passive smoking are also potential risk factors for GDM. However, because of the scope of our study and the differences in measuring ways and indicators, we were unable to study these variables.

## Prospects and conclusion

Future studies may focus on the relationship between exposure to different air pollutants before conception and GDM. The relationship between some other outdoor air pollutants, such as sulfur oxide, and GDM needs to be analyzed, and a dose–response manner should be of important consideration while analyzing the association of air pollutants with the risk of GDM. The effect of different combinations of air pollutants also needs to be studied more systematically. In addition, the distance from the main traffic road and noise are also potential risk factors for GDM,^49^ so as passive smoking during the pregnancy.^50^ Thus, further exploration for the effect of these factors is needed to help develop more accurate prevention strategies.

To sum up, the available evidence indicated direct association of air pollutants and GDM risk. High-quality and longitudinal studies are needed to improve our understanding of this association.
