# Supplementary material for: Air pollution and gestational diabetes mellitus: evidence from cohort studies
Source: BMJ Open Diabetes Res Care. 2020 Mar 18;8(1):e000937. doi: 10.1136/bmjdrc-2019-000937 (PMC7103802; doi:10.1136/bmjdrc-2019-000937)
Supplement: Supplementary data [file bmjdrc-2019-000937supp001.pdf]

Search strategies

Table S1. Data synthesized from included studies for meta-analysis.
Table S2. The risk bias assessment (Newcastle-Ottawa Quality Assessment Scale criteria) of studies included.

Figure. S1 Forest plot and pooled estimates of the association between exposure to BC, CO, $O_{3,}$ and PM_{10} with the risk of GDM.
Figure. S2 Funnel plot for the association between $PM_{2.5}$, O_3 and PM_{10} with the risk of GDM

Search strategies:

Pubmed：(All fields)

((((((((((((air pollution) OR air pollutant) OR particulate matter) OR PM 2.5) OR PM 10) OR nitrogen dioxide) OR O3) OR NO2) OR NOx) OR SO2) OR ozone) OR soot) OR smog) AND ((((((gestational diabetes) OR gestational diabetes mellitus) OR GDM) OR pregnancy diabetes mellitus) OR pregnancy diabetes) OR pregnancy glucose tolerance)

Embase: (Quick search)

(air pollution OR air pollutant OR particulate matter OR PM 2.5 OR PM 10 OR nitrogen dioxide OR O3 OR NO2 OR NOx OR SO2 OR ozone OR soot OR smog) AND (gestational diabetes OR gestational diabetes mellitus OR GDM OR pregnancy diabetes mellitus OR pregnancy diabetes OR pregnancy glucose tolerance)

Web of Science: (All fields)

(air pollution OR air pollutant OR particulate matter OR PM 2.5 OR PM 10 OR nitrogen dioxide OR O3 OR NO2 OR NOx OR SO2 OR ozone OR soot OR smog) AND (gestational diabetes OR gestational diabetes mellitus OR GDM OR pregnancy diabetes mellitus OR pregnancy diabetes OR pregnancy glucose tolerance)

Table S1. Data synthesized from included studies for meta-analysis.

source	pollutant	trimester	reported fully adjusted estimate (95% Cl)
Fleisch et al (2014) [1]	$PM_{2.5}$	second	central-site: 0.81 (0.62, 1.08) per 1.7 μg/m^3 of exposure<br>spatiotemporal: 0.94 (0.67, 1.34) per 2.0 μg/m^3 of exposure
	Black carbon	second	central-site: 0.69 (0.42, 1.13) per 0.16 μg/m^3 of exposure<br>spatiotemporal: 1.02 (0.73, 1.41) per 0.34 μg/m^3 of exposure
Fleisch et al (2016) [2]	$PM_{2.5}$	first	1.01 (0.93, 1.09) per 4.3 μg/m^3 of exposure
		second	0.97 (0.90, 1.05) per 4.5 μg/m^3 of exposure
Choe et al (2018) [3]	$PM_{2.5}$	first	1.02 (0.95, 1.09) per 1 IQR increase of exposure
		second	1.08 (1.00, 1.15) per 1 IQR increase of exposure
	Black carbon	first	1.03 (0.98, 1.08) per 1 IQR increase of exposure
		second	1.01 (0.97, 1.06) per 1 IQR increase of exposure
Hu et al (2015) [4]	$PM_{2.5}$	first	1.16 (1.11, 1.21) per 5 μg/m^3 of exposure
		second	1.15 (1.10, 1.20) per 5 μg/m^3 of exposure
	O_3	first	1.09 (1.07, 1.11) per 5 ppb
		second	1.12 (1.10, 1.14) per 5 ppb
Pedersen et al (2017) [5]	NO_2	first	common Danish: 0.89 (0.76, 1.03) per 10 μg/m^3 of exposure<br>WHO: 1.24 (1.03, 1.49) per 10 μg/m^3 of exposure
Pan et al (2017) [6]	O_3	first	0.97 (0.94, 0.99) per 1 ppb
		second	0.97 (0.94, 1.00) per 1 ppb
	SO_2	first	1.05 (1.00, 1.11) per 1 ppb
		second	1.06 (1.00, 1.12) per 1 ppb
	CO	first	1.08 (1.00, 1.15) per 0.1 ppm
		second	1.06 (0.99, 1.14) per 0.1 ppm
	NO_x	first	1.02 (1.00, 1.03) per 1 ppb
		second	1.01 (1.00, 1.03) per 1 ppb
	PM_{10}	first	0.99 (0.93, 1.05) per 10 μg/m^3 of exposure
		second	0.96 (0.90, 1.03) per 10 μg/m^3 of exposure
Malmqvist et al (2013) [8]	NO_x	second	Q1: 1.00 (reference)<br>Q2: 1.19 (0.99, 1.44)<br>Q3: 1.52 (1.28, 1.82)<br>Q4: 1.69 (1.41, 2.03)
Robledo et al 2014 [11]	$PM_{2.5}$	preconception	0.97 (0.93, 1.02) per 1 IQR increase of exposure
		first	0.98 (0.94, 1.03) per 1 IQR increase of exposure
	PM_{10}	preconception	0.99 (0.96, 1.02) per 1 IQR increase of exposure
		first	0.98 (0.95, 1.01) per 1 IQR increase of exposure
	NO_x	preconception	1.09 (1.04, 1.13) per 1 IQR increase of exposure
		first	1.06 (1.01, 1.10) per 1 IQR increase of exposure
	SO_2	preconception	1.05 (1.01, 1.09) per 1 IQR increase of exposure
		first	1.04 (1.00, 1.08) per 1 IQR increase of exposure
	CO	preconception	1.00 (0.97, 1.03) per 1 IQR increase of exposure
		first	0.99 (0.96, 1.03) per 1 IQR increase of exposure
	O_3	preconception	0.93 (0.90, 0.96) per 1 IQR increase of exposure
		first	1.00 (0.97, 1.03) per 1 IQR increase of exposure
Choe et al (2019) [12]	$PM_{2.5}$	first	0.97 (0.94, 1.00) per 1 IQR increase of exposure
		second	1.06 (1.03, 1.10) per 1 IQR increase of exposure
	NO_2	first	1.05 (1.01, 1.09) per 1 IQR increase of exposure
		second	1.02 (0.98, 1.06) per 1 IQR increase of exposure

Table S1. Continued.

Jo et al. (2019) [13]	NO_2	preconception	1.10 (1.07, 1.13) per 10.4 ppb
		first	1.02 (0.99, 1.05) per 10.4 ppb
	$PM_{2.5}$	preconception	1.04 (1.01, 1.06) per 6.5 μg/m^3 of exposure
		first	0.98 (0.95, 1.00) per 6.5 μg/m^3 of exposure
	PM_{10}	preconception	1.03 (1.00, 1.06) per 16.1 μg/m^3 of exposure
		first	0.98 (0.95, 1.01) per 10 μg/m^3 of exposure
	O_3	preconception	0.94 (0.92, 0.95) per 15.7 ppb
		first	0.95 (0.94, 0.97) per 15.7 ppb

Abbreviations: NO_2, nitrogen dioxide; $PM_{2.5}$, particulate matter ≤ 2.5 μm in diameter; PM_{10}, particulate matter ≤ 10 μm in diameter; NO_x, nitrogen oxides; O_3, ozone; SO_2, sulfur dioxide; BC, black carbon; CO, carbon monoxide; SD, standard deviation; IQR, interquartile range.

Table S2. The risk bias assessment (Newcastle-Ottawa Quality Assessment Scale criteria) of studies included.

**Study**	**Selection**				**Comparability**	**Outcome**			**Quality score**
	Representativeness of the exposed cohort	Selection of the non-exposed cohort from the same source as exposed cohort	Ascertainment of exposure	Demonstration that the outcome of interest was not present at the start of the study	Comparability of cohorts based on the design or the analysis	Ascertainment of outcome	Was follow-up long enough for outcomes to occur?	Adequacy of follow-up of cohorts	
Fleisch et al (2014) [1]	Participants were recruited from Harvard Vanguard Medical Associates, Boston, Massachusetts, USA	Yes«	Measured daily $PM_{2.5}$ and black carbon at a monitoring site located atop the Harvard University Countway Library in Boston, Massachusetts «	No	The study controls for Using age, race/ethnicity, education, household income, history of GDM in a previous pregnancy, family history of diabetes mellitus, smoking habits, and date of the last menstrual period updated with ultrasound, body mass index (BMI; kilograms per meter squared) from self-reported height and weight, gestational weight ««	Record linkage«	Yes«	Complete follow up all subject. «	7
Fleisch et al (2016) [2]	Participants registered live births in Massachusetts	Yes«	This model incorporates aerosol optical depth data from the MODIS Satellite, and classic land-use regression techniques to generate daily $PM_{2.5}$ exposure «	No	The study controls for age, race/ethnicity, education, smoking status, prenatal insurance, infant sex, date of birth directly, median annual household. «	Record linkage«	Yes«	Complete follow up all subject. «	7
Choe et al (2018) [3]	Participants were genuinely representative as they were from Women & Infants Hospital of Rhode Island, USA. «	Yes«	Estimated daily $PM_{2.5}$ at each maternal residential address using a spatial-temporal informed by both land-use regression and satellite remote sensing. «	No	The study controls for age, race, education, health insurance, tobacco use in pregnancy, and marital status. «	Record linkage«	Yes«	Complete follow up all subject. «	7

Table S2. Continued.

**Study**	**Selection**				**Comparability**	**Outcome**			**Quality score**
	Representativeness of the exposed cohort	Selection of the non-exposed cohort from the same source as exposed cohort	Ascertainment of exposure	Demonstration that the outcome of interest was not present at the start of the study	Comparability of cohorts based on the design or the analysis	Ascertainment of outcome	Was follow-up long enough for outcomes to occur?	Adequacy of follow-up of cohorts	
Hu et al (2015) [4]	Participants were genuinely representative as their reconds were from the Bureau of Vital Statistics and Office of Health Statistics and Assessment, Florida Department of Health. «	Yes«	Air pollution exposure data were obtained from the U.S. EPA and CDC's National Environmental Public Health Tracking Network. «	No	The study controls for gestational age, ethnicity , maternal education, smoking during pregnancy, the season of conception, prenatal care began residential area, median household income, and marital status. «	Record linkage«	Yes«	Complete follow up all subject. «	8
Pedersen et al. (2017) [5]	Women who participated in the Danish National Birth Cohort	Yes«	Written informed consent was obtained from all participants at enrollment. The Danish Data Protection Agency approved the present study. «	No	Basic adjusted models for maternal pre-pregnancy BMI and height, parity, maternal age and season of conception (LMP), maternal education and household disposable income Further adjustment for lifestyle factors included maternal smoking and physical activity and railway (dB) noise ««	Record linkage«	Yes«	72,745 women in the present study(72% of the source population)«	7
Pan et al (2017) [6]	Representative samples from the Birth Registration Database in Taiwan. «	Yes«	Air pollution data were collected from 66 stations located on the main the island of Taiwan and nearby participant's resident townships .«	No	The study controls for age, body mass index (BMI), weight gain during pregnancy, socioeconomic status (SES), tobacco, alcohol and betel use, parity, and fetal gender were used as covariates. ««	Record linkage«	Yes«	Complete follow up all subject. «	8

Shen et al (2017) [7]	Representative samples from Taiwan's National Health Insurance Research Data (NHIRD)«	Yes«	Air pollutant concentration data were obtained from the monitoring data supervised by the Environmental Protection Administration of Taiwan. «	No	The study controls for age, year at delivery, season of delivery, number of births, obesity, history of polycystic ovary syndrome(PCOS), and disease burden indicated by Charlson's Co-morbidity Index (CCI), personal monthly income and city/township specific median family income, and city/township level of urbanization ««	Record linkage«	Yes«	Complete follow up all subject. «	8

Table S2. Continued.

**Study**	**Selection**				**Comparability**	**Outcome**			**Quality score**
	Representativeness of the exposed cohort	Selection of the non-exposed cohort from the same source as exposed cohort	Ascertainment of exposure	Demonstration that the outcome of interest was not present at the start of the study	Comparability of cohorts based on the design or the analysis	Ascertainment of outcome	Was follow-up long enough for outcomes to occur?	Adequacy of follow-up of cohorts	
Malmqvist et al. (2013) [8]	Women who had singleton deliveries at The Swedish Medical Birth Registry in Sweden. «	Yes«	Information on NOx in Scania from an emission database (EDB)Road traffic data were obtained from the Swedish National Road Database «	No	Adjusted analyses of preeclampsia for significant risk factors including prepregnancy body mass index (BMI), smoking habits at first antenatal visit, ethnicity, parity, type 1 diabetes, gestational diabetes, and maternal age. «	Record linkage«	Yes«	Complete follow up all subject. «	8
Lu et al (2015) [9]	Women who underwent a two-step approach for GDM diagnosis and who gave birth at the Department of Obstetrics and Gynecology of DMF-CYCH, in southwestern Taiwan «	Yes«	Air pollutant concentrations were obtained from the Chiayi station, a nearby fixed-site monitoring station operated by Taiwan Environmental Protection Administration (EPA)«	No	The study controls for age, weight , height , parity, body mass index, season, moving averages of temperature, and relative humidity. «	Record linkage«	Yes«	Complete follow up all subject. «	7

van den Hooven et al (2009) [10]	Mothers and children of different ethnicities living in Rotterdam, the Netherlands. Ideally, enrolment in the study took place in early pregnancy. «	Yes«	Individual traffic exposure estimates at each participant's home address were assessed using Geographical Information Systems (GIS)«	No	The study controls for maternal age at intake, maternal educational level, maternal ethnicity, maternal body mass index (BMI), parity, maternal smoking, maternal alcohol consumption, and fetal sex. «	Record linkage«	Yes«	Complete follow up all subject. «	8

Table S2. Continued.

Study	Selection				Comparability	Outcome			Quality score
	Representativeness of the exposed cohort	Selection of the non-exposed cohort from the same source as exposed cohort	Ascertainment of exposure	Demonstration that the outcome of interest was not present at the start of the study	Comparability of cohorts based on the design or the analysis	Ascertainment of outcome	Was follow-up long enough for outcomes to occur?	Adequacy of follow-up of cohorts	
Robledo et al (2015) [11]	Participants were genuinely representative as they were from 12 clinical centers (with 19 hospitals) across 15 hospital referral regions. «	Yes«	The Air Quality and Reproductive Health (AQRH) study linked pregnancies from the CSL to air pollutant exposures estimated using a modified Community Multi-scale Air Quality Model(CMAQ) version 4.7.1. «	No	The study controls for maternal age, race/ethnicity, parity, marital status, insurance status, hospital type, prenatal history of smoking, alcohol use. «	Record linkage«	Yes«	Complete follow up all subject. «	8

Choe et al (2019)[12]	Linked birth certificate files to hospital discharge data provided by the New York State Department of Health Statewide Planning and Research Cooperative System to verify medical conditions before and during pregnancy.	Yes«	Air pollutant concentrations were obtained from the New York City Community Air Survey (NYCCAS)«	No	The study controls for age, ethnicity, education, parity, working during pregnancy, conception year, and deprivation index.	Record linkage«	Yes«	Complete follow up all subject. «	8
Jo et al. (2019)[13]	KPSC covers Imperial, Kern, Los Angeles, Orange, Riverside, San Bernardino, San Diego, San Luis Obispo, Santa Barbara, and Ventura counties, with 14 medical center service areas.	Yes«	Monthly averages for each pollutant between 1998 and 2009 were obtained from data compiled from the EPA regional air quality monitoring network across Southern California.	No	The study controls for age, race, education, annual household income, and prepregnancy body mass index. «	Record linkage«	Yes«	Complete follow up all subject. «	8

Abbreviations: $PM_{2.5}$, particulate matter ≤ 2.5 μm in diameter; GDM, gestational diabetes mellitus; EPA, Environmental Protection Agency; CDC, Centers for Disease Control; GIS, Geographical Information Systems; EDB, emission database; BMI, body mass index; NHIRD, National Health Insurance Research Data; AQRH, Air Quality, and Reproductive Health; CMAQ, Community Multi-scale Air Quality Model; SES, socioeconomic status; PCOS, polycystic ovary syndrome; CCI, Charlson's Co-morbidity Index.

Figure. S1 Forest plot and pooled estimates of the association between exposure to BC, CO, O_3, and PM_{10} with the risk of GDM.

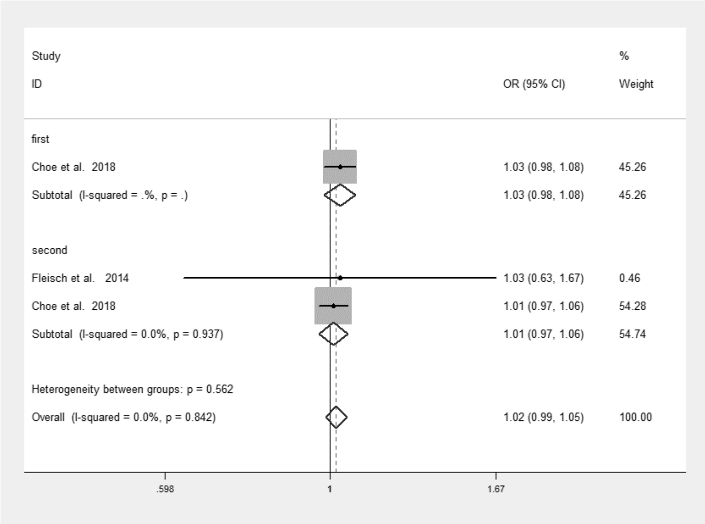

A. BC and risk of GDM

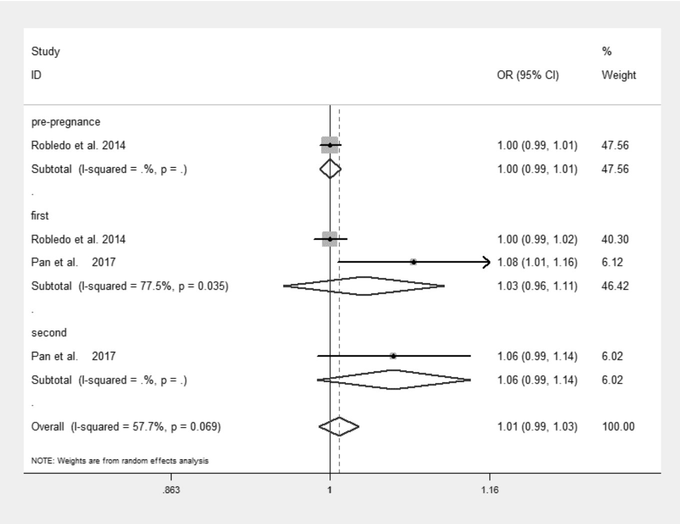

B. CO and risk of GDM

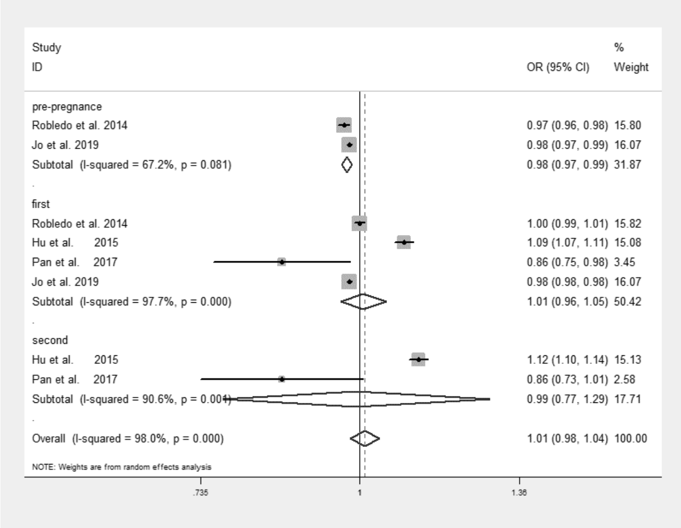

C. O_3 and risk of GDM

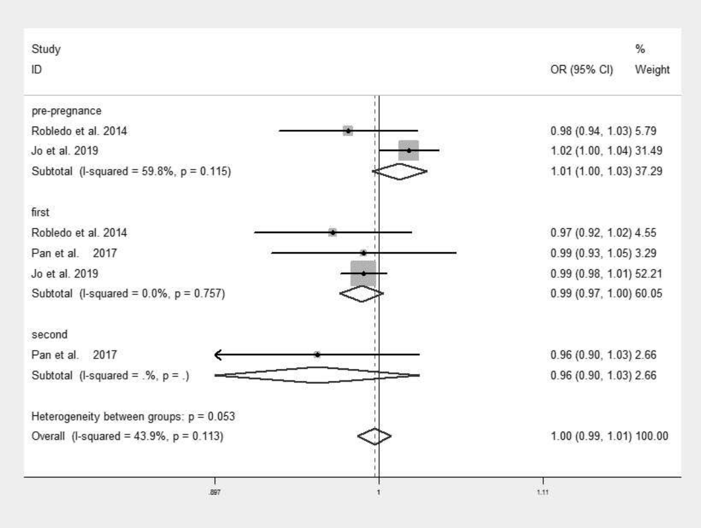

D. PM_{10} and risk of GDM

Abbreviations: GDM, gestational diabetes mellitus; OR, odds ratio; BC, black carbon; CO, carbon monoxide; O_3, ozone; PM_{10}, particulate matter ≤ 10 μm in diameter

pre-pregnancy, the exposure to air pollutants was measured before pregnancy; first, the exposure to air pollutants was measured during the first trimester; second, the exposure to air pollutants was measured during the second trimester.

Figure. S2 Funnel plot for the association between $PM_{2.5}$, O_3 and PM_{10} with the risk of GDM

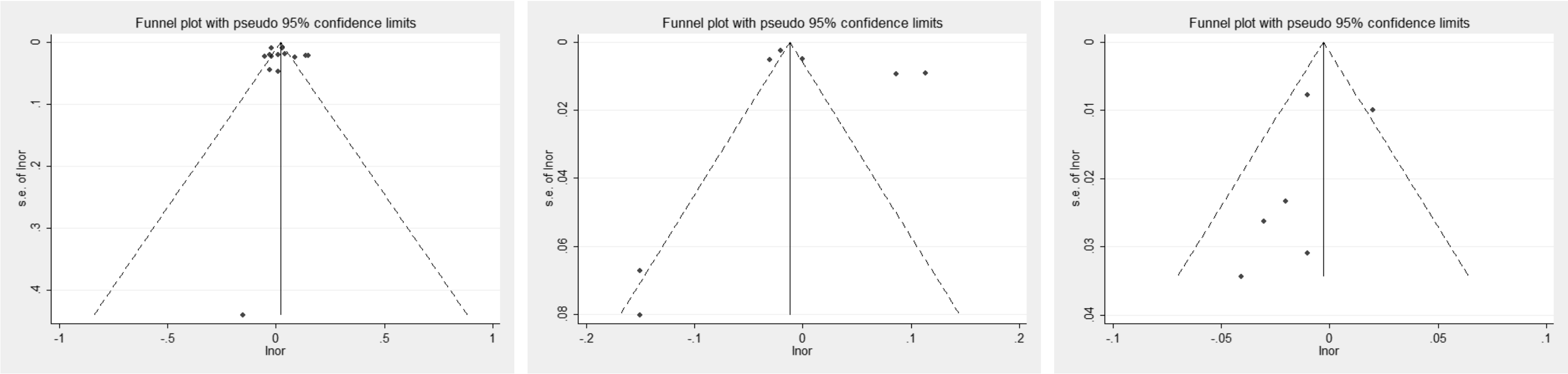

A. Funnel plot for $PM_{2.5}$ and risk of GDM. B. Funnel plot for O_3 and risk of GDM. C. Funnel plot for PM_{10} and risk of GDM.

Abbreviations: GDM, gestational diabetes mellitus; $PM_{2.5}$, particulate matter ≤ 2.5 μm in diameter; O_3, ozone; PM_{10}, particulate matter ≤ 10 μm in diameter.

References:

[1] Fleisch A F, Gold D R, Rifas-Shiman S L, et al. Air pollution exposure and abnormal glucose tolerance during pregnancy: the project Viva cohort[J]. Environ Health Perspect, 2014, 122(4): 378-83.

[2] Fleisch A F, Kloog I, Luttmann-Gibson H, et al. Air pollution exposure and gestational diabetes mellitus among pregnant women in Massachusetts: a cohort study[J]. Environ Health, 2016, 15: 40.

[3] Choe S A, Kauderer S, Eliot M N, et al. Air pollution, land use, and complications of pregnancy[J]. Sci Total Environ, 2018, 645: 1057-1064.

[4] Hu H, Ha S, Henderson B H, et al. Association of Atmospheric Particulate Matter and Ozone with Gestational Diabetes Mellitus[J]. Environ Health Perspect, 2015, 123(9): 853-9.

[5] Pedersen M, Olsen S F, Halldorsson T I, et al. Gestational diabetes mellitus and exposure to ambient air pollution and road traffic noise: A cohort study[J]. Environ Int, 2017, 108: 253-260.

[6] Pan S C, Huang CC, Lin S J, et al. Gestational diabetes mellitus was related to ambient air pollutant nitric oxide during early gestation[J]. Environ Res, 2017, 158: 318-323.

[7] Shen H N, Hua S Y, Chiu C T, et al. Maternal Exposure to Air Pollutants and Risk of Gestational Diabetes Mellitus in Taiwan[J]. Int J Environ Res Public Health, 2017, 14(12).

[8] Malmqvist E, Jakobsson K, Tinnerberg H, et al. Gestational diabetes and preeclampsia in association with air pollution at levels below current air quality guidelines[J]. Environ Health Perspect, 2013, 121(4): 488-93.

[9] Lu MC, Wang P, Cheng T J, et al. Association of temporal distribution of fine particulate matter with glucose homeostasis during pregnancy in women of Chiayi City, Taiwan[J]. Environ Res, 2017, 152: 81-87.

[10] Van Den Hooven EH, Jaddoe V W, De Kluizenaar Y, et al. Residential traffic exposure and pregnancy-related outcomes: a prospective birth cohort study[J]. Environ Health, 2009, 8: 59.

[11] Robledo C A, Mendola P, Yeung E, et al. Preconception and early pregnancy air pollution exposures and risk of gestational diabetes mellitus[J]. Environ Res, 2015, 137: 316-22.

[12] Choe S A, Eliot M N, Savitz D A, et al. Ambient air pollution during pregnancy and risk of gestational diabetes in New York City[J]. Environ Res, 2019, 175: 414-420.

[13] Jo H, Eckel SP, Chen J C, et al. Associations of gestational diabetes mellitus with residential air pollution exposure in a large Southern California pregnancy cohort[J]. Environ Int, 2019, 130: 104933.
